# Evaluation of Large Language Models for Structured Data Extraction From Interstitial Lung Disease Clinical Notes: Comparative Study

**DOI:** 10.2196/90547

**Published:** 2026-06-26

**Authors:** Stephanie Ji Chen, Manoj Venkat Maddali, Curtis Langlotz, Christian Bluethgen, Jonathan Chen, Rishi Raj

**Affiliations:** 1 Division of Pulmonary, Allergy, and Critical Care Medicine Stanford Medicine Stanford, CA United States; 2 Department of Biomedical Data Science Stanford Medicine Stanford, CA United States; 3 Division of Pulmonary and Critical Care Medicine Piedmont HealthCare Atlanta, GA United States; 4 Department of Radiology and Center for Artificial Intelligence in Medicine and Imaging Stanford Medicine Stanford, CA United States

**Keywords:** electronic health records, information extraction, interstitial lung diseases, large language models, natural language processing

## Abstract

**Background:**

Most clinically relevant data are in unstructured clinical notes, which are verbose and imprecise, making structured data extraction a costly bottleneck for screening patients for studies or maintaining health care registries. This challenge is particularly pronounced in interstitial lung disease (ILD) and requires significant human effort to interpret notes and determine classification to create an ILD registry. Large language models (LLMs) have the potential to significantly reduce this cost and effort.

**Objective:**

We aim to compare the performance of various LLMs for structured data extraction from unstructured ILD clinic notes. Our primary aim was to evaluate LLM extraction of binary structured data (yes/no answers) from clinical notes regarding key ILD clinical questions. A secondary analysis evaluated select LLMs for the extraction of multiclass data to determine ILD classification.

**Methods:**

We used 12 different LLMs to extract binary answers to 10 ILD clinical questions from the most recent clinic notes of 100 ILD clinic patients. We additionally used 2 LLMs (gpt-oss-20b and gpt-oss-120b) to extract multiclass data regarding ILD classification. Prompts were created with the assistance of ChatGPT (OpenAI) and refined with an iterative approach by testing on a prompt engineering cohort of 10 ILD clinic patient notes. Ground truth was established by consensus among 3 ILD physicians. LLM performance was evaluated using accuracy, precision, recall, and *F*_1_-scores.

**Results:**

LLMs processed each interface call of a clinical note-prompt combination in 1-2 seconds, with estimated costs ranging from less than US $0.001 to US $0.11 (or approximately US $0.05 to US $10.50 per clinical note accounting for 10 runs and 10 binary prompts) depending on the model. Out of the 12 LLMs assessed, 7 models (Claude 3.5 Sonnet [Anthropic], GPT-4o, gpt-oss-20b, gpt-oss-120b, o1, o1-mini, and o3-mini [OpenAI]) performed at human-level accuracy, similar to that of the 3 ILD clinicians (96.2%). A total of 5 LLMs performed significantly worse than humans (Holm-adjusted *P*≤.003 for all). gpt-oss-120b, o1, and o3-mini models achieved the highest *F*_1_-scores of all the evaluated LLMs. There was no significant difference in model accuracy among the top tier models (Claude 3.5 Sonnet, gpt-oss-20b, gpt-oss-120b, o1, o1-mini, and o3-mini), though GPT-4o achieved significantly lower accuracy than o1 (Bonferroni-adjusted *P*=.04). Multiclass data extraction using gpt-oss-120b and gpt-oss-20b demonstrated lower accuracy when compared to its corresponding binary data extraction (91.1% and 88.0%, respectively). There was no significant difference in accuracy between gpt-oss-120b and gpt-oss-20b for multiclass extraction.

**Conclusions:**

Multiple LLMs consistently achieved human-level accuracy in extracting structured binary data from ILD clinical notes, while being orders of magnitude faster and cheaper. Multiclass data extraction was possible but associated with a lower accuracy. LLMs are promising tools that can be used for clinical data extraction to improve clinical research efficiency.

## Introduction

Most clinically relevant data are contained in unstructured text such as clinical notes [1]. Consequently, clinical research often entails significant human effort and cost to review and extract binary data from unstructured text [2,3]. This challenge is particularly pronounced in interstitial lung disease (ILD), where clinical documentation is prone to verbosity and imprecision. Reliable and faithful extraction of structured data by large language models (LLMs) can significantly reduce the cost and effort currently required to screen patients for studies or create and maintain ILD registries or databases.

LLMs are machine learning models designed to predict the next word or token in a sequence based on their training data [4]. Introduction of the transformer architecture in 2017 enabled LLMs to analyze whole sentences or paragraphs simultaneously for relationships, rather than process a single word at a time. This significantly increased the size of LLMs and the data they could be trained on [5]. The extensive data and parameters leveraged by the transformer architecture have enabled LLMs to create rich, multilayered mathematical representations of language, which contribute to the LLM’s appearance of intelligence [6]. When given a prompt, these transformer models generate text by predicting the most probable next word based on training data. This process continues iteratively until coherent sentences and paragraphs are produced in response to the prompt.

**Figure 1:**
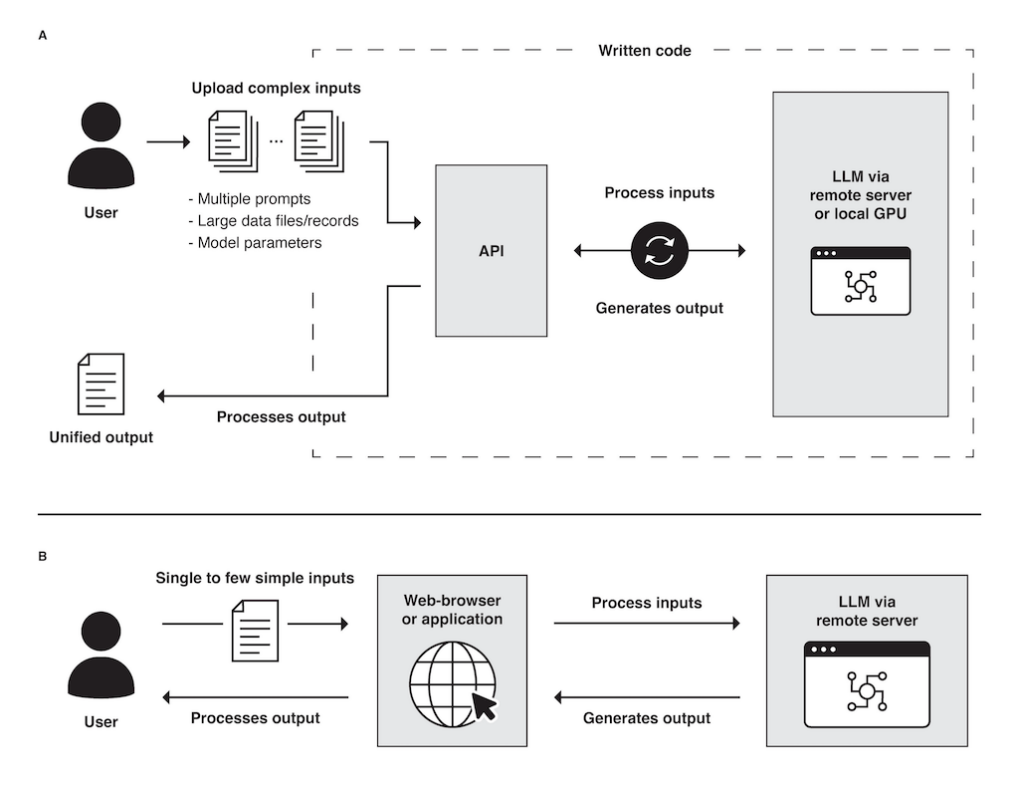
Schematic representation of accessing large language models (LLMs). (A) The user programs the application programming interface (API), which can encode multiple records/data, prompts/queries, and model parameters. This is then sent to the LLM either via remote server or locally on a graphics processing unit (GPU) machine, which generates responses that are processed by the API into a unified response and sent back to the user. (B) The user provides a single query to a web browser or application, which accesses the LLM via a remote server. The LLM then generates a single response and sends it back to the user.

The most common way to access available LLMs is through a web or app-based interface. The user submits a prompt or query through the LLM web page or application on a device and receives a response, which is displayed in the browser or application. This approach, though convenient, is limited by its inability to adjust model parameters or process more than one document at a time. An alternative for interacting with LLMs involves using an application programming interface (API). This approach involves writing and executing code on a local machine that takes user inputs such as prompts, records, and desired model parameters, and sends them to the LLM via the API for processing. The LLM then generates a response, which is returned to the user and can be further processed to the desired format (Figure 1). By accessing LLMs via an API, the user can process large sets of queries and data sequentially without requiring individual input as in the web-based approach. An additional advantage of this method is the potential to modify various model parameters that are otherwise hidden or fixed in a web or app-based interface, such as the temperature, which determines the stochasticity (“deterministic” vs “creative”) of the model responses [7].

To use LLMs, well-crafted prompts are essential. Prompt engineering is the process of crafting effective and precise instructions or questions as inputs for LLMs to produce the desired output or answer. For example, a simple prompt such as “Does the patient have ILD?” is likely to generate nonspecific answers due to a lack of context and specificity. In such cases, providing additional context and clarification in the prompts is essential to achieve accurate responses.

This study was designed as a proof of concept to assess the performance characteristics of current LLMs in extracting structured binary data (ie, yes or no answers) from clinical notes for patients with ILD regarding key ILD clinical questions. The focus of our study was not to address nuances in ILD classification or to develop all the necessary prompts for a registry, but rather to determine whether LLMs can reliably and faithfully extract binary data from verbose and ambiguous documentation when given a specific set of instructions. Additionally, we also performed a secondary exploratory analysis to assess the feasibility of multiclass extraction using 2 open-weight models of differing sizes. Some of the results of these studies have been previously reported in the form of an abstract [8].

## Methods

### Cohort

**Figure 2:**
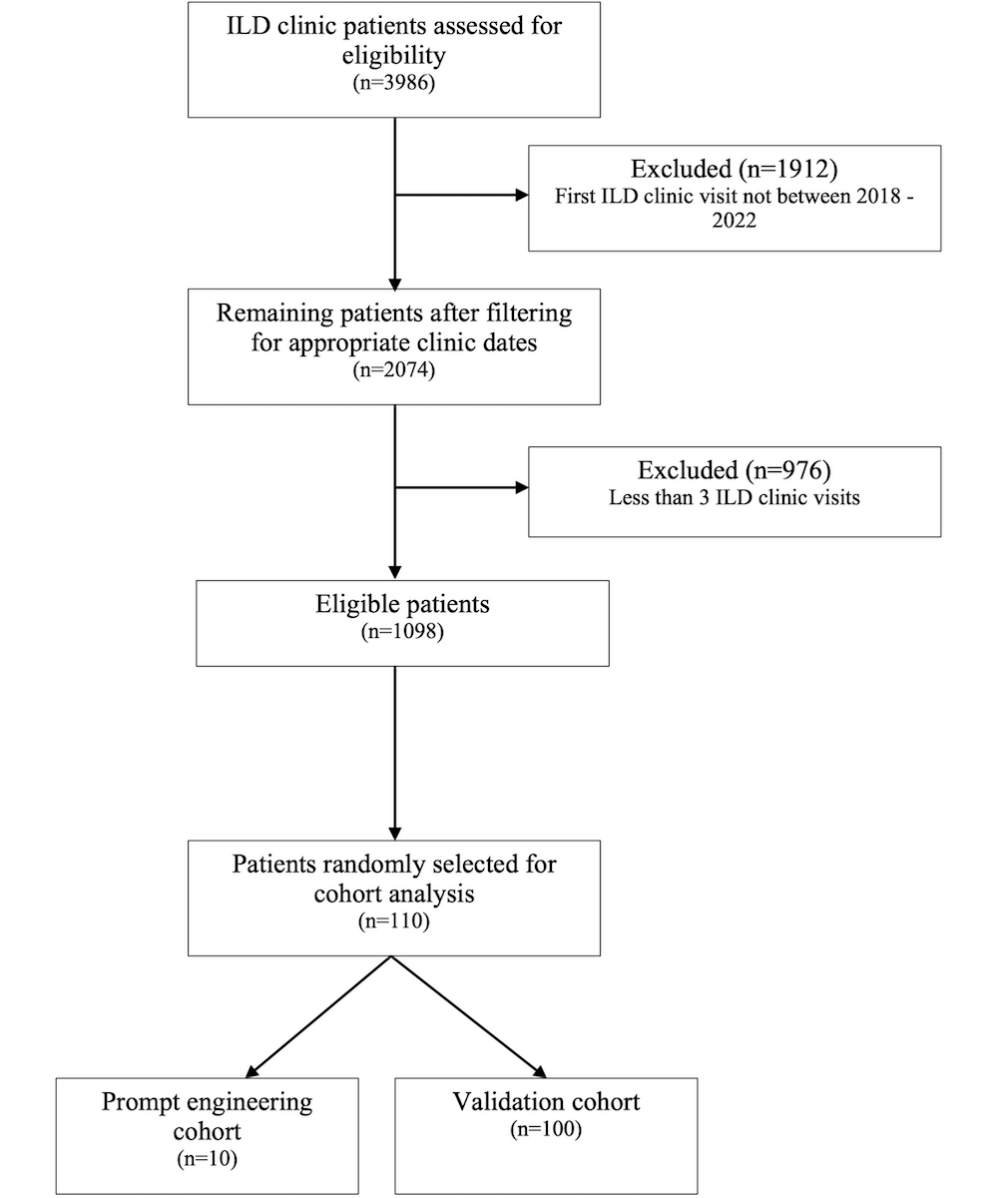
Flowchart of prompt engineering and large language model evaluation cohorts. ILD: interstitial lung disease.

We identified patients who were initially seen in the Stanford Interstitial Lung Disease Clinic between 2018 and 2022 and had at least 3 ILD clinic visits; this was to ensure adequate follow-up visits to reach a diagnosis. From this cohort, 10 patients and 100 patients were randomly selected for the prompt engineering cohort and LLM evaluation validation cohort, respectively (Figure 2). These cohorts were kept separate. The most recent ILD clinic note available for each patient was used for this study.

### Ethical Considerations

This study was approved by the Institutional Review Board of Stanford University (IRB-83821). A waiver of informed consent was obtained, given that this retrospective chart review study involved no more than minimal risk and does not adversely affect the rights and welfare of the participants. All data used in the study were stored securely by investigators, without sharing of Health Insurance Portability and Accountability Act (HIPAA) information to outside investigators or sources. No identifying patient information has or will be published for the purposes of this study. No compensation was provided for this study. No interventions, clinical recruitment, or patient-level interactions were conducted.

### Prompt Engineering Process

An iterative approach was used to create prompts to extract binary (yes or no) responses from clinic notes for the 10 patients in the prompt engineering cohort. We identified 10 clinical questions related to ILD classification, treatment, and progression. Prompts were initially created in a simple question format and tested using GPT-4o (OpenAI) on the prompt engineering cohort. Given poor performance with simple prompts, chain-of-thought and heuristic prompts were developed for each clinical question following prior published methods on prompt engineering [9,10]. We used ChatGPT (OpenAI) to help create the chain-of-thought and heuristic prompts based on the simple format prompts. Chain-of-thought prompts instructed models to reason step-by-step through relevant clinical criteria before producing a final yes/no output (eg, “Please create a chain-of-thought prompt to determine if a patient has ILD, reasoning through the relevant clinical features before giving a final yes or no answer.”). Heuristic prompts detailed specific instructions and constrained the model to output only a binary answer (eg, “Please create a heuristic prompt to determine if a patient has ILD, answering only yes or no.”). Heuristic prompts demonstrated the highest accuracy when tested on the prompt engineering cohort with GPT-4o and selected as the final prompt format. Based on a review of incorrect responses in the prompt engineering cohort, an ILD physician iteratively refined the prompts by adjusting the rules accordingly (eg, adding missed definitions, incorporating negative constraints to prevent over-classification, or clarifying ambiguous terminology from notes) and retesting prompts until 100% accuracy was achieved in the prompt engineering cohort. This process took an estimated 3-5 iterations, depending on the prompt. The final heuristic prompts were applied uniformly to all 12 LLMs without model-specific modifications for validation, to allow for direct cross-model comparison under identical prompting conditions. Sample representations of prompts from initial creation to final iteration are shown in Textbox 1.

**Sample interstitial lung disease clinical note binary extraction prompts from initial creation to final version.**

**Initial prompts**

- Does the patient have interstitial lung disease (ILD) as explicitly stated in the note? Answer only “yes” or “no.”
- Has the patient had any respiratory-related hospitalizations based on the note? Respiratory-related hospitalizations are defined as any hospitalization due to increase in pulmonary symptoms. Answer only “yes” or “no.”

**Final prompts**

- Does the patient have interstitial lung disease (ILD) as explicitly stated in the note? Answer only “yes” or “no.” Keep the following rules in mind:
- Answer Yes if any of the following are explicitly mentioned in the note:

i. Diagnosis of interstitial lung disease (ILD), even if the type is unclassified or pending further work-up (eg, “ILD of uncertain etiology” is considered an ILD)

ii. Diagnosis of idiopathic pulmonary fibrosis (IPF)

iii. Diagnosis of sarcoidosis or sarcoid

iv. Diagnosis of pulmonary fibrosis or fibrotic lung disease

v. Presence of specific CT scan findings such as reticulation, honeycombing, traction bronchiectasis, fibrosis, usual interstitial pneumonia (UIP), nonspecific interstitial pneumonia (NSIP), or hypersensitivity pneumonitis (HP)

vi. Pathology showing ILD, fibrosis, UIP, NSIP, or HP

vii. Patient is on nintedanib (Ofev) or pirfenidone (Esbriet)

- Answer No if:

i. None of the above diagnoses, findings, or medications are mentioned in the note

ii. The diagnosis is explicitly ruled out (eg, No evidence of ILD)

iii. Patient has bronchiectasis without evidence of associated ILD

iv. ILD is not suggested or ruled out, and no related findings are present in the note

- If there is any discordance between different sections of the note (eg, a tentative diagnosis like possible sarcoidosis in the HPI but definitive diagnosis in the assessment and plan section), always use the diagnosis stated in the assessment and plan section to guide your answer
- Has the patient had a respiratory related hospitalization based on the note? Answer only “Yes” or “No.” Keep the following rules in mind:
- Answer Yes if any hospitalization or admission is mentioned in the note and is due to any of the following criteria:

i. Respiratory symptoms (eg, cough, shortness of breath/dyspnea, and increased or new oxygen requirement)

ii. Exacerbation of underlying lung disease

iii. Respiratory infection or illness

iv. Pleural effusion

v. Pneumothorax

vi. Volume overload

vii. Left or right sided heart failure (CHF, CHF exacerbation, HFrEF or HFpEF exacerbation)

- Answer No if:

i. There are no hospitalizations mentioned in the note (Note: emergency room or urgent care visits do not count as a hospitalization)

ii. Any hospitalization mentioned does not meet any of the criteria listed above

An additional multiclass response prompt for ILD classification was derived directly from the binary heuristic prompts regarding ILD diagnosis, and a separate iterative process was not undertaken. A complete list of final binary and multiclass prompts used for validation is shown in section S1 in Multimedia Appendix 1.

### Protected Health Information Compliance

HIPAA-compliant business associate agreements (BAAs) were signed with OpenAI, Anthropic, and Google to ensure appropriate handling and safeguarding of data prior to transmission, permitting the use of protected health information (PHI) in accordance with HIPAA Privacy and Security rules. Clinical notes were transmitted via API under these BAAs without complete deidentification prior. However, additional preprocessing using regular expressions to remove patient names, date of birth, and medical record numbers was performed as an added precautionary measure.

### LLM Information

We used a HIPAA-compliant pipeline to access the following LLMs: GPT-3.5 Turbo, GPT-4, GPT-4o, GPT-4o mini, o1, o1-mini, o3-mini (OpenAI), Claude 3.5 Sonnet, Claude 3.7 Sonnet (Anthropic), and Gemini 2.0 Pro (Google) [11-19]. We also ran open-weight reasoning models gpt-oss-20b and gpt-oss-120b (OpenAI) models locally on a HIPAA-compliant local graphics processing unit (GPU) machine [20].

Commercial models were accessed through 2 Stanford Health Care institutional gateways: the API Management gateway [21] for GPT-3.5 Turbo, GPT-4, GPT-4o, GPT-4o mini, o1-mini, and Claude 3.5 Sonnet; and the AI Hub gateway [22] for o1, o3-mini, and Claude 3.7 Sonnet. These gateways proxy requests to Azure OpenAI (GPT and o-series) and Amazon Web Services Bedrock (Claude) backends, respectively.

Gemini 2.0 Pro was accessed directly via the Google Vertex AI software development kit (project som-nero-phi-rishiraj-ild-rr, region us-central1). The specific model version used was the experimental build gemini-2.0-pro-exp-02-05, as this was the only Gemini 2.0 Pro variant available on Vertex AI at the time of data extraction. For Anthropic Claude models, the Bedrock model Amazon Resource Names include version-pinning dates (eg, claude-3-5-sonnet-20241022-v2:0 or claude-3-7-sonnet-20250219-v1:0). For OpenAI models accessed through the institutional gateways, the gateway routes requests by deployment alias (eg, gpt-4o or o1); the underlying model snapshot version is managed by the institution and is not returned in the API response, which is an inherent limitation of managed Azure OpenAI deployments. The exact API deployment identifiers, full end point URLs, and access protocols for each model evaluated in this study are summarized in Table S1 in Multimedia Appendix 2.

Both gpt-oss-120b and gpt-oss-20b were obtained from their official HuggingFace repositories (openai/gpt-oss-120b, commit b5c939de8f754692c1647ca79fbf85e8c1e70f8a; openai/gpt-oss-20b, commit 6cee5e81ee83917806bbde320786a8fb61efebee) with no fine-tuning or modifications applied to the released weights. Inference was performed using vLLM 0.10.1+gptoss on NVIDIA H100 NVL GPUs. gpt-oss-120b was served as a single instance with tensor parallelism (TP) across 2 GPUs (TP=2), while gpt-oss-20b was served as 2 independent instances (TP=1, 1 per GPU, ports 8000 and 8001). Both models used fp16 KV cache, max-model-len of 65,536, max-num-seqs of 32, and max-num-batched-tokens of 8192, with chunked prefill, prefix caching, and async scheduling enabled. GPU memory use was set to 0.90. The complete inference configuration is summarized in Table S2 in Multimedia Appendix 2.

### Clinical Data Extraction Through the LLM Pipeline

Each note-binary prompt combination in the validation cohort (which was kept strictly separate from the prompt engineering cohort) was processed through the models 10 times to assess consistency of the LLMs, with each run analyzed separately. Therefore, each model had 10,000 inference calls for binary clinical data extraction based on 100 notes, 10 binary prompts, and 10 runs. In addition, each note-multiclass prompt combination was processed through the 2 open-weight models, gpt-oss-20b and gpt-oss-120b 10 times, thus an additional 1000 inference calls for these 2 models. These models were selected specifically for multiclass clinical data extraction because they share the same underlying architecture, differing only in parameter count, allowing for an assessment of how model scale influences performance for more complex extraction tasks. The temperature parameter for GPT-3.5 Turbo, GPT-4, GPT-4o, GPT-4o mini, Claude 3.5 Sonnet, Claude 3.7 Sonnet, and Gemini 2.0 Pro was set at 0.0 to maximize reproducibility [23]. Claude 3.7 Sonnet was evaluated with extended thinking disabled.

For the OpenAI reasoning models accessed via the commercial API (o1, o1-mini, and o3-mini), the temperature parameter was fixed at 1.0 and could not be modified by the user. The reasoning_effort parameter, which controls the number of internal reasoning tokens generated before producing a response, was not explicitly set for these models; per OpenAI’s API documentation, this defaulted to “medium.” The o1-mini model does not support the reasoning_effort parameter. Similarly, the open-weight reasoning models gpt-oss-20b and gpt-oss-120b temperature parameter was also fixed at 1.0 and not modified by the user.

Commercial and cloud-hosted models (GPT-3.5 Turbo, GPT-4, GPT-4o, GPT-4o mini, o1, o1-mini, o3-mini, Claude 3.5 Sonnet, Claude 3.7 Sonnet, and Gemini 2.0 Pro) were evaluated between February and March 2025. The 2 open-weight models (gpt-oss-20b and gpt-oss-120b) were evaluated after their public release in August 2025.

### Reference Standard for Ground Truth

A total of 3 ILD clinicians independently reviewed the same clinical notes and provided responses based on the prompts. They then convened to reach a consensus answer for each note-prompt combination, which served as the ground truth against which to score the LLMs. The scorers were blinded to each other’s and LLM responses during individual scoring, and to LLM responses during the consensus meeting.

### Statistical Analysis

Accuracy for each LLM model and for each of the ILD clinicians was determined by comparing their responses to the ground truth and reported as the mean across the 10 runs per patient. Precision, recall, and *F*_1_-score were calculated for each LLM. For the multiclass analysis, model outputs were mapped to ILD categories using the following rule: any response containing “unclassifiable” was assigned to the unclassifiable category (reflecting clinical practice in which the unclassifiable designation takes precedence even when specific subtypes are also under consideration); otherwise, the first-listed class was taken as the primary diagnosis. Macro- and weighted-averaged precision, recall, and *F*_1_-score were computed across the ILD categories. Consistency between runs for a given model was assessed by calculating the percent agreement of the majority prediction for each note-prompt combination. Interrater reliability among the 3 ILD physician responses was measured using the Fleiss κ statistic [24]. For McNemar pairwise comparisons, the majority-vote consensus prediction across 10 runs was taken as each model’s response. Differences in model accuracy were analyzed using the Cochran *Q* test followed by post hoc pairwise comparisons conducted using the McNemar test with Bonferroni correction for multiple comparisons (78 pairs; corrected α=.00064). Differences in model accuracy to human accuracy were conducted using the McNemar test with the Holm-Bonferroni step-down procedure (family of 12 comparisons; family-wise α=.05) [25,26]. Differences between LLM models in the multiclass task were assessed using the McNemar test on majority-vote consensus predictions and an independent-samples *t* test on per-run macro-*F*_1_. Statistical analysis was performed in Python. In the absence of established sample size guidelines for LLM evaluation, a cohort of 100 patients was deemed to be an appropriate size for this proof-of-concept study.

## Results

Of the 3986 ILD clinic patients assessed for eligibility, 2888 were excluded for not meeting the inclusion criteria. Of the eligible 1098 patients, 110 patients were randomly selected, 10 for prompt engineering and 100 for LLM validation. A flow diagram of cohort selection is shown in Figure 2.

The median word count for the ILD clinical notes was 1788 (IQR 1214-2344) words. Each LLM took approximately 1-2 seconds to process a clinical note-prompt combination. Models were accessed via API with rate limits of up to 30,000,000 tokens/minute (eg, GPT-4o), allowing for efficient parallel processing of clinical notes (1 token is approximately equal to 1 word). Pricing varied across models, with estimated API costs ranging from less than US $0.001 to US $0.11 per clinical note-prompt combination. Accounting for 10 binary prompts per note and 10 repeated runs per binary prompt-note combination, estimated total processing costs ranged from approximately US $0.05 to US $10.50 per clinical note depending on the model used (see section S2 in Multimedia Appendix 1 for detailed cost breakdown for LLMs),

OpenAI’s gpt-oss-120b and o1 achieved the highest accuracy at 97.5% (95% CI 97.3%-97.7%) and 97.3% (95% CI 97.1%-97.4%), respectively. The mean accuracy of the 3 ILD physicians was 96.2% (95% CI 92.3%-100.0%). Cochran *Q* test demonstrated statistically significant overall differences in accuracy across models (*Q*=1437.45, *df*=12; *P*<.001). There was no statistically significant difference in accuracy between the ILD physicians and 7 of the models: Claude 3.5 Sonnet, GPT-4o, o1, o1-mini, o3-mini, gpt-oss-20b, and gpt-oss-120b.

Among these top-performing models, there was no significant difference in accuracy from each other except between o1 and GPT-4o*.* The other 5 models (GPT-4, GPT-4o-mini, GPT-3.5 Turbo, Claude 3.7 Sonnet, and Gemini 2.0 Pro) had significantly lower accuracy compared to ILD physicians. GPT-3.5 Turbo was the worst-performing model, with significantly lower accuracy than every other model. Mean accuracy for each model, along with the ILD physicians, is shown in Figure S1 in Multimedia Appendix 3. Pairwise McNemar test results comparing model accuracy and pairwise McNemar test results comparing model accuracy to human accuracy are shown in Tables S1 and S2, respectively, in Multimedia Appendix 4.

Prompt 8, asking whether a patient has sarcoidosis, performed the best of all the prompts with nearly 100% accuracy for all models except for GPT-3.5 Turbo (accuracy 45.2%). Prompt 9, asking about progression of respiratory symptoms, in general, had the worst accuracy of all the prompts, ranging from 80%-90% accuracy. A detailed breakdown of each model’s mean accuracy over 10 runs by prompt is shown in Table S3 in Multimedia Appendix 4*.*

The precision, recall, and *F*_1_-score for each model averaged over their 10 runs are shown in Table 1*.* OpenAI’s gpt-oss-120b, o1, and o3-mini models achieved the highest *F*_1_-scores of all the evaluated LLMs.

**Table 1:** Average accuracy, precision, recall, and F1-scores over 10 runs for each large language model for binary clinical data extraction.

Model	Accuracy, % (95% CI)	*F*_1_-score	Precision	Recall
gpt-oss-120b	97.5 (97.3-97.7)	0.962	0.955	0.970
o1	97.3 (97.1-97.4)	0.958	0.969	0.948
o3-mini	97.2 (97.0-97.3)	0.957	0.962	0.953
gpt-oss-20b	97.0 (96.7-97.2)	0.955	0.944	0.965
o1-mini	96.6 (96.4-96.8)	0.948	0.960	0.937
Claude 3.5 Sonnet	95.5 (95.3-95.6)	0.931	0.950	0.913
GPT-4o	95.5 (95.3-95.6)	0.930	0.957	0.903
GPT-4	94.4 (94.4-94.5)	0.931	0.915	0.948
GPT-4o-mini	94.2 (94.1-94.3)	0.909	0.946	0.875
Claude 3.7 Sonnet	92.0 (91.8-92.2)	0.870	0.959	0.796
Gemini 2.0 Pro	90.6 (90.4-90.7)	0.850	0.895	0.811
GPT-3.5 Turbo	70.2 (70.0-70.3)	0.681	0.535	0.938

All models with temperature 0.0 achieved close to 100% agreement over 10 runs. The reasoning models with temperature 1.0 had percent agreement ranging from 93%-100%. A detailed breakdown of each model’s mean percent agreement by prompt is shown in Table S4 in Multimedia Appendix 4. The Fleiss κ for the 3 ILD physicians was 0.88, indicating a high level of agreement.

For the multiclass prompt evaluation, precision, recall, and *F*_1_-scores as macro and weighted averages (mean and SD) across the 7 ILD categories are reported in Table 2. Multiclass prompts had lower accuracy compared with binary prompts at 91.1% (vs 97.5%) and 88.0% (vs 97.0%) for gpt-oss-120b and gpt-oss-20b, respectively. gpt-oss-120b model demonstrated significantly higher macro-*F*_1_ across runs (independent-samples *t* test, t_18_=2.93; *P*=.009) compared to gpt-oss-20b and approximately half the run-to-run variability of the 20b model. Patient-level comparison using the McNemar test revealed no significant difference in accuracy between the 2 models (0.91 vs 0.88; McNemar *P*>.99), with 89 of 100 patients classified concordantly correct. Per-class *F*_1_-score was ≥0.80 in all 7 ILD categories for both models. Of note, both models achieved perfect per-class *F*_1_-scores (1.00) for idiopathic pulmonary fibrosis (n=17) and sarcoidosis (n=4), with small class size a possible contributor when interpreting the results. A detailed breakdown of per-class *F*_1_-score, precision, and recall is shown in Table S5 in Multimedia Appendix 4.

**Table 2:** Precision, recall, and F1-scores as macro and weighted averages for multiclass response prompt. Values are mean and SD across 10 runs (n=100 patients per run).

Metric	gpt-oss-120b, mean (SD)	gpt-oss-20b, mean (SD)
Accuracy	0.911 (0.014)	0.880 (0.028)
Macro precision	0.926 (0.009)	0.907 (0.022)
Macro recall	0.924 (0.015)	0.897 (0.023)
Macro-F1	0.923 (0.012)	0.901 (0.020)
Weighted F1	0.913 (0.013)	0.889 (0.024)

## Discussion

### Principal Findings

In this proof-of-concept study, we demonstrate that multiple transformer-based LLMs can reliably and efficiently extract unstructured data from ILD clinical notes into binary data. With appropriate prompt engineering, 7 of the 12 tested models consistently achieved high accuracy over various ILD prompts, similar to that of ILD clinicians (96.2%), while being orders of magnitude faster and cheaper. Additionally, these models all demonstrated excellent performance with an *F*_1_-score of >0.9. High precision, recall, and *F*_1_-scores are critical for appropriate LLM use in clinical research when using it to screen patients for studies or to create and maintain ILD registries or databases. High precision reduces false positives, preventing the inclusion of ineligible patients that can introduce bias and skew research results. High recall reduces false negatives, ensuring that all relevant patients are included in the study, thus preserving the statistical power of the study. A high *F*_1_-score ensures a strong balance between precision and recall.

Interestingly, we noted that there was degradation in performance in 2 of the newer LLMs, Claude 3.7 Sonnet and Gemini 2.0 Pro (both released in February 2025), compared to the older models Claude 3.5 Sonnet (released June 2024) and GPT-4o (released May 2024). This finding could reflect a tension between the model training or alignment and the strict prompt output constraints. The newer LLMs undergo increased layers of training and reinforcement learning from human feedback [18]. While this allows models to perform more complex reasoning tasks and provide elaborate responses, this may paradoxically reduce their ability to follow constrained instructions, such as “answer only yes or no,” as specified in our binary prompts. Prior work has shown that LLMs are vulnerable to negative or distractor requirements [27,28]. Our findings may reflect a related but distinct phenomenon where LLMs become vulnerable following instruction requirements that do not align with their pretraining. However, we do note that Claude 3.7 Sonnet was evaluated with extended thinking disabled, and thus the degradation observed in that model cannot be attributed to its extended reasoning mechanism. Instead, it appears to stem from changes in the underlying base model’s training and alignment. The role of extended reasoning in constrained-instruction performance remains an open question that this study was not designed to address. Regardless, it is likely important to tailor model selection to the clinical task and recognize that newer, more advanced models are not necessarily better for all tasks.

A qualitative analysis at the prompt level was performed to better understand differences in prompt accuracy across models and human assessors. Prompt 8, asking whether a patient has sarcoidosis, had the best accuracy for all models and among human assessors. One possible reason is that sarcoidosis is generally diagnosed by biopsy, which, if positive, is usually unambiguous and likely to be clearly documented in clinical notes. In contrast, other ILDs may carry lower diagnostic confidence and are often documented with multiple differential diagnoses. In contrast, prompt 9, assessing progression of respiratory symptoms, had the worst accuracy. This is likely due to the vague and often contradictory documentation of symptoms. For example, multiple clinical notes would document increased cough in the *history* section, but in the *assessment and plan* section, indicate “clinically stable” or “no changes in respiratory symptoms.” “Carrying forward” text from previous notes contributed to many of these inconsistencies, which led to confusion on how to answer the prompt and greater disagreements among the human assessors. The difference in prompt accuracies highlights how the quality of clinical note documentation impacts clinical data extraction that is not solely attributable to LLM deficiencies. Therefore, while using and assessing LLMs, it is also important to consider the underlying source from which they are extracting information.

LLMs with temperature 0.0 had close to 100% agreement for each clinical note-prompt combination, demonstrating high consistency in their outputs with repeated runs. In contrast, the reasoning models with temperature 1.0 had a wider range in agreement. Therefore, in certain clinical data extraction tasks where reproducibility is paramount, choosing a model with an adjustable temperature parameter to 0.0 may be preferable without sacrificing accuracy. Furthermore, given the near-deterministic behavior observed with a temperature of 0.0, a single API call per note would likely be sufficient for clinical deployment.

As a secondary exploratory analysis, multiclass data extraction was assessed using the locally deployed gpt-oss-20b and gpt-oss-120b models*.* Multiclass extraction resulted in lower accuracy and macro-*F*_1_ for ILD classification compared with binary extraction using the same model (gpt-oss-20b: accuracy 88.0% vs 97.0%; macro-*F*_1_ 0.901 vs 0.955; gpt-oss-120b: accuracy 91.1% vs 97.5%; macro-*F*_1_ 0.923 vs 0.962). Although there was no significant difference in accuracy between the 2 models for multiclass prompts (McNemar *P*>.99), gpt-oss-120b demonstrated significantly higher macro-*F*_1_ across runs (*P*=.009) and approximately half the run-to-run variability compared to gpt-oss-20b. This suggests that the increased model scale primarily confers higher consistency rather than peak accuracy in the multiclass task. Similarly, binary extraction also demonstrated minimal differences in accuracy and computational efficiency across model capacities (excluding GPT-3.5 Turbo). Furthermore, the top-performing standard instruction-tuned models (Claude 3.5 Sonnet and GPT-4o) demonstrated no significant difference in accuracy compared to the reasoning models (o1, o1-mini, o3-mini, gpt-oss-20b, and gpt-oss-120b), despite the latter using extended chain-of-thought inference at substantially higher computational cost. This suggests that for structured clinical extraction tasks, whether binary or more complicated multiclass tasks, more advanced or larger LLM architectures may not confer substantial benefit. Rather, the prompts are more important for success in clinical extraction tasks. Accordingly, for structured binary data extraction from clinical notes, smaller and older models are likely to offer a more favorable cost-to-benefit ratio without sacrificing performance.

In addition to lower accuracy, multiclass extraction produced greater variability in model responses, requiring additional postprocessing to standardize outputs for analysis. This variability primarily had 2 forms: inconsistent use of abbreviations and class names (eg, “HP” vs “hypersensitivity pneumonitis”) and production of ranked differential diagnoses rather than single-class labels (eg, output: “unclassifiable, idiopathic pulmonary fibrosis, HP”). The latter raises a methodological question of how such outputs should be graded and requires careful consideration of labeling rules prior to analysis. In our study, we treated any response containing “unclassifiable” as an unclassifiable prediction, reflecting clinical practice in which this designation takes precedence over specific subtype considerations during multidisciplinary discussion. These findings highlight the increased variability of multiclass extraction, placing a greater burden on clinical researchers to correctly interpret and normalize data extraction results, which can reduce the efficiency gains of multiclass extraction compared with binary extraction.

Given that LLMs are designed to predict the next word based on their training data rather than retrieve information from a database, hallucinations are inevitable [29]. Hallucinations occur when LLMs generate plausible outputs that are factually incorrect. However, they can be mitigated through model training and thoughtful prompt engineering. Simple prompts, which asked a direct question without additional detail, performed poorly across all models. In contrast, heuristic prompts, which provided explicit rules, enabled multiple LLMs to accurately extract binary data regarding ILD classification, treatment, and progression. These findings are consistent with prior studies on prompt engineering [9,10]. Thoughtful prompt engineering was crucial to obtain desired clinical data extraction results from LLMs and mitigate some of the limitations stemming from vague or contradictory clinical note documentation. However, as noted above, we suspect that prompt constraints may have contributed to performance degradation in newer models such as Gemini 2.0 Pro. Thus, prompt engineering should be tailored to both the specific LLM and the clinical task at hand to obtain the best performance, rather than a generic prompt that may not optimize performance across all models. While our prompts were developed and validated on ILD clinical notes from a single center, limiting direct generalizability of our specific prompts to other clinical specialties and institutions, the underlying prompt engineering methodology of developing heuristic prompts with explicit clinical rules is broadly applicable.

Our study showed that human assessment also failed to achieve 100% accuracy; in fact, there was a wider range in accuracy among the 3 ILD physicians compared to most of the evaluated LLMs. The human errors were the result of failing to follow the prompt instructions or failing to pick up information hidden within the verbose note. With increased volume of patients and/or length of clinical notes to review, the potential for human error is often likely to become more pronounced and can greatly depend on the individual chosen for evaluation. In contrast, LLM performance and efficiency are not affected by volume.

We believe that our proof-of-concept study has meaningful implications for improving clinical workflows, especially in clinical research. Manual structured clinical data extraction from clinical notes represents a major bottleneck for clinical research and creating patient data registries; it is time-consuming and labor-intensive. Furthermore, it is prone to human error. Use of LLMs would enable automated data extraction for disease-specific patient registry creation (eg, ILD registries) and cohort selection for clinical and quality improvement research (eg, identifying patients with idiopathic pulmonary fibrosis for a study), at a fraction of the time and cost required with traditional chart review. It would also free up clinicians and researchers to focus their efforts on developing higher-order clinical and scientific questions for the advancement of knowledge, rather than on the tedious process of chart review and data abstraction. The availability and high accuracy of open-weight models such as gpt-oss-20b and gpt-oss-120b further expand implementation possibilities, as these models can be locally housed within an institution’s infrastructure without the need to transmit PHI data to an external API, greatly decreasing risks of privacy concerns.

While we demonstrate that multiple LLMs can be effectively leveraged for clinical data extraction, translating this success for use in clinical research practice requires key operational steps. First, a robust and secure infrastructure pipeline is required to ensure PHI compliance when using LLMs. User inputs and prompts sent to LLMs on remote servers (eg, GPT series models from OpenAI, Claude series models from Anthropic, and Gemini series models from Google) are stored and often used to further train these models; however, entities owning and running these LLMs do not provide specific details regarding this process. These LLMs, therefore, cannot be used without appropriate safeguards to process PHI-containing data. While we used regular expressions during the preprocessing of input notes to remove patient names, date of birth, and medical record numbers as an added precautionary measure, this is not sufficient for PHI compliance, given the brittle nature of regular expressions when applied to unstructured narrative clinical notes. Options to process PHI data using these LLMs include either running smaller models locally on a computer with adequate GPU memory (eg, the Llama series models by Meta) or signing a HIPAA-compliant BAA with providers such as OpenAI or Anthropic. The HIPAA-compliant BAA allows for “zero retention,” where the data sent to the LLMs are handled appropriately with safeguarding of PHI, not used to train models, and are HIPAA compliant in other respects as well [30,31]. We ensured that all LLMs used in our study were HIPAA compliant, with BAAs in place. Second, careful and thoughtful prompt engineering is required. This involves testing and refining prompts on a representative set of clinical notes to determine that LLMs are providing the desired output. Third, performance characteristics of the final prompts need to be validated on the data they will be used on before incorporating them into the research pipeline.

### Comparison to Prior Work

This study builds on prior work by evaluating multiple LLMs and comparing their performance. Prior studies largely compared limited number of models for clinical data extraction, while we systematically compared 12 models spanning different developers and model generations including standard instruction-tuned models (eg, GPT-4 and Claude 3.5 Sonnet)*,* reasoning models (o1, o1-mini, and o3-mini), and open-weight reasoning models that can be deployed locally (gpt-oss-20b and gpt-oss-120b) [32]. Furthermore, we compared LLM performance to a human benchmark (in our case, ILD specialists), which is the current gold standard for clinical data extraction. Additionally, by using ILD clinic notes, we extend upon previous studies that largely focused on structured or semistructured reports such as radiology or pathology reports [33]. ILD notes are often complex and narrative, containing multiple or conflicting diagnoses. However, we were able to demonstrate that multiple LLMs could still faithfully extract structured clinical information with clear instructions. Notably, this was achieved without fine-tuning of models. Liu et al [34] demonstrated that fine-tuning of open-source models was required to achieve consistent human-level performance for pathology extraction tasks; however, fine-tuning requires machine learning expertise that many clinicians are not well-versed in. In contrast, our study demonstrates that multiple commercial and open-weight LLMs can achieve human-level accuracy across ILD clinical extraction tasks through prompt engineering alone without the need for fine-tuning, thus reducing the technical barrier for clinician research adoption. Finally, our comparison of binary vs multiclass extraction offers further methodological insight for clinical data extraction. We demonstrate that multiclass extraction is feasible, although it is associated with a loss of accuracy compared with binary extraction. While binary extraction achieved higher accuracy, the reduction in accuracy with multiclass extraction may be acceptable given gains in extraction practicality and reduction of required queries, depending on the intended clinical or research use of the extracted data.

### Limitations and Further Work

Our study had several limitations due to runtime considerations and effort cost. First, our prompt engineering cohort was small and randomly selected, limiting our ability to capture edge cases and further refine prompts. This led to some inaccurate outputs that could have easily been adjusted in the prompts. We suspect that a larger, more variable prompt engineering cohort can further enhance the performance of LLMs in binary and multiclass extraction. Second, our prompts were iteratively refined using GPT-4o and uniformly applied to all models. We recognize that this inherently leads to bias toward GPT models. Furthermore, our prompts were zero-shot prompts and restricted models to “answer only yes or no.” As a result, standard instruction-tuned models are at a disadvantage compared to reasoning models, given their inability to internally reason and perform chain-of-thought processes. Prompts optimized for one model family may not elicit the same quality of responses from other model families, and the relative performance gaps should be interpreted with caution, with the understanding that prompts may not have been optimized for the specific model. Additionally, our multiclass prompt used for secondary analysis did not undergo the same iterative refinement as our binary prompts and may contribute to the lower performance scores in multiclass extraction compared to binary extraction. For clinical implementation, model-optimized prompts should be used for the best results. Third, our validation cohort was small and limited to a single clinic and institution. Multi-institutional studies would help to determine whether a standardized prompt library could be developed for ILD clinical data extraction more broadly. Further research should assess LLM performance with well-crafted prompts across a broader range of clinic notes and institutions to better evaluate generalizability across different specialties and note types. However, given that multiple models were noninferior to humans in accuracy despite using complex, narrative ILD notes, we hypothesize that with appropriate prompt engineering, high accuracy comparable to human-level accuracy would be maintained across other clinical settings. Fourth, the same ILD physicians who determined the consensus reference standard were graded on their individual responses to determine the accuracy of the human assessments. This leads to artificial inflation of human performance. While this represents a methodological limitation, it does not undermine our findings in this proof-of-concept study that multiple LLMs are able to achieve human-level accuracy. Rather, if anything, the limitation is conservative with respect to our primary findings. Assessing human accuracy with an independent adjudicator would likely narrow the performance gap between humans and LLMs further and may show additional models that also achieve human-level accuracy. Fifth, while interrater agreement among the 3 ILD physicians to establish ground truth was high (Fleiss κ=0.88), this still reflects disagreement among physicians for 10%-12% of cases. Given that disagreements were resolved by internal consensus rather than an independent adjudicator, this introduces the risk of convened consensus bias, where the final label may reflect social or hierarchical dynamics of the 3 physicians, rather than a purely objective clinical standard. Future work using an independent fourth reader as a tiebreaker or formal Delphi-style anonymous resolution can help reduce the influence of group dynamics on the reference standard against which both humans and LLMs are evaluated. Sixth, we were unable to adjust the temperature parameter for the reasoning models, thus o1, o1-mini, and o3-mini had a fixed temperature of 1.0 compared to the other models which we had adjusted temperature to 0.0. This may lead to some variations in comparison of model performance although prior studies have shown that changes in temperature from 0.0 to 1.0 did not have significant effect on problem solving performance of LLMs [23]. Seventh, we did not adjust for other model parameters such as top_p or top_k. It is possible that adjustment of these parameters or a different combination of parameters, may improve clinical data extraction, especially for the older models. Eighth, the multiclass prompts did not undergo a rigorous iterative refinement process like that of the binary prompts, as it was derived directly from the binary classification prompts and meant to be a preliminary feasibility assessment. Thus, multiclass accuracy may improve with further prompt refinement in future studies. Finally, our study was designed as a proof-of-concept for structured binary and limited multiclass extraction from clinical notes. We did not evaluate more complex clinical information extraction tasks, such as temporal reasoning across notes, relation extraction, or patient-level aggregation of information from multiple encounters. Given the rapid pace of LLMs’ evolution, these represent critical areas for future exploration.

### Conclusions

Our findings demonstrate that multiple LLMs can consistently achieve human-level accuracy in extracting structured binary data from unstructured ILD clinical data, while being orders of magnitude faster and cheaper. This was able to be accomplished relying only on prompt engineering and without fine-tuning of models prior to usage. Multiclass extraction is also feasible, although it is associated with a reduction in accuracy.

## Data Availability

The datasets generated or analyzed during this study are not publicly available due to patient privacy protections and institutional restrictions under the Health Insurance Portability and Accountability Act.

## Appendix

Multimedia Appendix 1 Final prompts selected for large language model (LLM) evaluation and LLM processing cost estimation.

Multimedia Appendix 2 Deployment identifiers and inference configuration parameters for locally deployed open-weight models (gpt-oss-20b and gpt-oss-120b), including application programming interface end points, HuggingFace repository commits, graphics processing unit hardware specifications, and vLLM serving settings.

Multimedia Appendix 3 Mean accuracy of large language models (LLMs) and interstitial lung disease physician assessors with 95% CI. Dark gray fill represents no statistically significant difference in accuracy between humans and LLMs.

Multimedia Appendix 4 Tables for mean accuracy, pairwise McNemar test results comparing model accuracy, pairwise McNemar test results comparing large language model’s accuracy to human accuracy, and percent agreement breakdown by model and prompt.

## Abbreviations

API: application programming interface
BAA: business associate agreement
GPU: graphics processing unit
HIPAA: Health Insurance Portability and Accountability Act
ILD: interstitial lung disease
LLM: large language model
PHI: protected health information
TP: tensor parallelism
