# Supplementary material for: Evaluation of Large Language Models for Structured Data Extraction From Interstitial Lung Disease Clinical Notes: Comparative Study
Source: J Med Internet Res. 2026 Jun 26;28:e90547. doi: 10.2196/90547 (PMC13354945; doi:10.2196/90547)
Supplement: Multimedia Appendix 1 [file jmir_v28i1e90547_app1.docx]

**S1: Final prompts selected for large language model evaluation.**

CT = computed tomography; HPI = history of present illness; IVIG = intravenous immunoglobulin; CHF = congestive heart failure; HFrEF = heart failure with reduced ejection fraction; HFpEF = heart failure with preserved ejection fraction

*Binary Prompts:*

General instructions: You are a medical assistant that reads notes from patient charts and answers questions. Please read the following patient note and answer the questions based on the information explicitly stated within the note and the question context. Answer only ‘Yes’ or ‘No’, do not provide an explanation for your answer.

Question 1: Does the patient have interstitial lung disease (ILD) as explicitly stated in the Note? Answer only ‘Yes’ or ‘No’. Keep the following rules in mind:

- 1. Answer Yes if any of the following are explicitly mentioned in the Note:
     1. Diagnosis of interstitial lung disease (ILD), even if the type is unclassified or pending further work-up (e.g. ‘ILD of uncertain etiology’ is considered an ILD)
     2. Diagnosis of idiopathic pulmonary fibrosis (IPF)
     3. Diagnosis of sarcoidosis or sarcoid
     4. Diagnosis of pulmonary fibrosis or fibrotic lung disease
     5. Presence of specific CT scan findings such as: reticulation, honeycombing, traction bronchiectasis, fibrosis, usual interstitial pneumonia (UIP), non-specific interstitial pneumonia (NSIP), or hypersensitivity pneumonitis (HP)
     6. Pathology showing ILD, fibrosis, UIP, NSIP, or HP
     7. Patient is on nintedanib (Ofev) or pirfenidone (Esbriet)
  2. Answer No if:
     1. None of the above diagnoses, findings, or medications are mentioned in the note
     2. The diagnosis is explicitly ruled out (e.g. ‘No evidence of ILD’)
     3. Patient has bronchiectasis without evidence of associated ILD
     4. ILD is not suggested or ruled out, and no related findings are present in the note
  3. If there is any discordance between different sections of the Note (e.g., a tentative diagnosis like ‘possible sarcoidosis’ in the HPI but definitive diagnosis in the assessment and plan section), always use the diagnosis stated in the assessment and plan section to guide your answer

Question 2: Does the patient have idiopathic pulmonary fibrosis (IPF) as explicitly stated in the Note? Answer only ‘Yes’ or ‘No’. Keep in mind the following rules:

- 1. Answer Yes if:
     1. IPF is explicitly mentioned as the primary or most likely diagnosis being considered, even if it is described as ‘likely secondary to’ or ‘probably caused by’
     2. IPF is the most likely diagnosis, even if additional work-up is needed
  2. Answer No if:
     1. Another form of ILD (e.g. connective tissue disease related ILD or CTD-ILD, hypersensitivity pneumonitis) is considered more likely than IPF
     2. There are multiple ILD diagnoses being considered (e.g IPF vs hypersensitivity pneumonitis)
     3. Diagnosis in the note is unclassified or unclassifiable (e.g. ‘pulmonary fibrosis of unclear etiology’)
     4. Further evaluation or work-up is required to confirm IPF (such as pending imaging or biopsy), unless IPF is already strongly suspected based on clinical or radiological findings.
  3. If there is any discordance between different sections of the note (e.g. ‘possible IPF’ in the HPI but IPF is confirmed in the assessment and plan), always use the diagnosis stated in the assessment and plan section to guide your answer

Question 3: Does the patient have pulmonary fibrosis or fibrotic lung disease? Answer only ‘Yes’ or ‘No’. Keep the following rules in mind:

- 1. Answer Yes if any of the following are explicitly mentioned in the note:
     1. Diagnosis of idiopathic pulmonary fibrosis (IPF)
     2. Diagnosis of fibrotic lung disease or pulmonary fibrosis (even if not progressive)
     3. Definitive CT scan findings consistent with pulmonary fibrosis, which include:
        1. Honeycombing
        2. Reticulation (including any form, e.g. reticular opacities or changes)
        3. Presence of fibrosis on CT scan
     4. Pathology findings showing fibrosis, UIP or usual interstitial pneumonia, or fibrosing non-specific interstitial pneumonia or NSIP (Note: Non-fibrosing NSIP would not count)
     5. Patient is on nintedanib (Ofev) or pirfenidone (Esbriet)
  2. Answer No if:
     1. None of the above diagnosis, findings, or medications are explicitly mentioned in the note
     2. The note explicitly rules out pulmonary fibrosis entirely (e.g. ‘No fibrosis present’)
  3. If there is any discordance between different sections of the note (e.g. ‘possible IPF’ in the HPI but IPF is confirmed in the assessment and plan), always use the diagnosis stated in the assessment and plan section to guide your answer

Question 4: Is the patient currently on the anti-fibrotics nintedanib (Ofev) or pirfenidone (Esbriet) at this current visit? Answer only ‘Yes’ or ‘No’. Keep the following rules in mind:

- 1. Answer Yes if the Note explicitly states the patient is currently taking nintedanib (Ofev) or pirfenidone (Esbriet) during this visit
  2. Answer No if:
     1. The note does not mention either medication
     2. The note only mentions that the patient is planning to start or is recommended to start one of the anti-fibrotics (e.g. “start Ofev”)
     3. The patient was previously on one of these medications, but they have been discontinued without mention of restarting or initiating an anti-fibrotic medication (nintedanib/Ofev or pirfenidone/Esbriet)

Question 5: Is the patient currently on any of the following immunosuppressive medications at this current visit: prednisone, methotrexate, azathioprine/Imuran, mycophenolate/myfortic/cellcept, cyclophosphamide/Cytoxan, rituximab, IVIG, tacrolimus, tocilizumab/Actemra, or tofacitinib/Xeljanz? Answer only ‘Yes’ or ‘No’. Keep the following rules in mind:

- 1. Answer Yes if the note explicitly states the patient is currently taking any of the listed immunosuppressive medications at the time of this visit (e.g., "patient is on prednisone," "patient is prescribed methotrexate").
  2. Answer No if:
     1. None of the listed medications are mentioned in the note
     2. The note only mentions that the patient is planning to start or is recommended to start one of the listed medications (e.g., "starting prednisone," "recommend starting tocilizumab").
     3. Any of the listed medications were mentioned but have been discontinued with no indication to restart or start a new medication from the list

Question 6: Does the patient have a connective tissue disease associated interstitial lung disease (CTD-ILD) as explicitly stated in the Note? Answer only ‘Yes’ or ‘No’. Keep the following rules in mind:

- 1. Answer Yes if all of the following are true:
     1. The note explicitly states a definitive diagnosis of a connective tissue disease, which include mixed CTD (mCTD), rheumatoid arthritis (RA), Sjogren’s, systemic sclerosis or scleroderma (SSc), lupus (SLE), vasculitis (e.g. ANCA), or myositis (including dermatomyositis, anti-synthetase syndrome, polymyositis, or inflammatory myositis). This would also include diagnosis of CTD, even if exact type is not specific (e.g. confirmed CTD-ILD)
     2. The note explicitly states a definitive diagnosis of ILD. (Note: bronchiectasis alone is Not considered an ILD)
     3. There are no other ILD diagnoses being considered (e.g. idiopathic pulmonary fibrosis, hypersensitivity pneumonitis). This means no competing diagnosis like IPF, hypersensitivity pneumonitis, or any other ILD should be listed as primary consideration
  2. Answer No if:
     1. Any of the above criteria are not satisfied
     2. CTD-ILD is described as a secondary possibility (e.g. ‘scleroderma associated ILD cannot be ruled out’) or another diagnosis (e.g. other forms of pulmonary fibrosis or ILD) is more likely. Note: If the ILD is explicitly described as being caused by the CTD, such as ‘NSIP secondary (2/2) RA-ILD’', this should not be considered a ‘secondary possibility’ but a confirmed diagnosis of CTD-ILD
     3. Diagnosis in the note is unclassified or unclassifiable (e.g. ‘unclassifiable ILD’)
     4. Further evaluation or work-up is required to confirm the presence of a CTD (e.g. pending autoimmune work-up) unless the diagnosis of CTD is already established and the additional tests are for disease activity or treatment monitoring
  3. If there is any discordance between different sections of the note (e.g. ‘possible scleroderma’ in the HPI but SSc-ILD is confirmed in the assessment and plan), always use the diagnosis stated in the assessment and plan section to guide your answer

Question 7: Does the patient have hypersensitivity pneumonitis (HP) as explicitly stated in the Note? Answer only ‘Yes’ or ‘No’. Keep the following rules in mind:

- 1. Answer Yes if:
     1. Hypersensitivity pneumonitis (HP) is the primary or most likely diagnosis being considered in the note. This means HP must be explicitly mentioned in the assessment and plan section as the definitive or the most probable diagnosis.
     2. HP is the most likely cause of the lung disease (e.g. ‘pulmonary fibrosis secondary to hypersensitivity pneumonitis’)
  2. Answer No if:
     1. HP is described as a secondary possibility (e.g. ‘HP cannot be ruled out’) or another diagnosis (e.g. CTD-ILD, IPF) is being considered.
     2. Diagnosis in the Note is unclassified or unclassifiable (e.g. ‘pulmonary fibrosis of unclear etiology’)
     3. Further evaluation or work-up is required to confirm HP (such as bronchoscopy or biopsy), unless HP is already strongly suspected based on clinical or radiological findings.
  3. If there is any discordance between different sections of the note (e.g. ‘possible HP’ in the HPI but HP is confirmed in the assessment and plan), always use the diagnosis stated in the assessment and plan section to guide your answer

Question 8: Does the patient have sarcoidosis or sarcoid as explicitly stated in the Note? Answer only ‘Yes’ or ‘No’. Keep the following rules in mind:

- 1. Answer Yes if:
     1. Sarcoidosis/sarcoid is the primary or most likely diagnosis being considered in the note. This means sarcoid must be explicitly mentioned in the assessment and plan section as the definitive or the most probable diagnosis.
     2. Sarcoid is the most likely cause of the lung disease (e.g. ‘pulmonary Nodules secondary to sarcoid’)
  2. Answer No if:
     1. Sarcoid is described as a secondary possibility (e.g. ‘sarcoid cannot be ruled out’) or another diagnosis (e.g. hypersensitivity pneumonitis) is being considered.
     2. Diagnosis in the Note is unclassified or unclassifiable (e.g. ‘pulmonary fibrosis of unclear etiology’)
     3. Further evaluation or work-up is required to confirm sarcoidosis (such as bronchoscopy or biopsy), unless sarcoid is already strongly suspected based on clinical or radiological findings.
  3. If there is any discordance between different sections of the Note (e.g. ‘possible sarcoid’ in the HPI but sarcoid is confirmed in the assessment and plan), always use the diagnosis stated in the assessment and plan section to guide your answer

Question 9: Does the patient have progression in respiratory symptoms at this current visit? Answer only ‘Yes’ or ‘No’. Keep the following rules in mind:

- 1. Answer Yes if all of the following are true:
     1. Any mention of patient having worsening respiratory symptoms at the current visit, defined as any of the following:
        1. Increased or worsening cough, sputum production, or new hemoptysis or coughing with blood
        2. Increased shortness of breath or dyspnea
        3. Decreased ability to exercise given shortness of breath
        4. New oxygen requirement or need for increased liters of oxygen (e.g. was stable on 3L oxygen, now requiring 5L)
        5. Greater drop in oxygen saturations at rest or with exertion compared to prior
  2. Answer No if:
     1. Any of the above criteria are not satisfied
     2. Symptoms are stable or improved at the current visit, even if they were worsening previously
     3. There is no new evidence or mention of worsening symptoms at the current visit (e.g. ‘stable respiratory symptoms’). Note: if the note indicates ‘stable respiratory symptoms but new cough or increased sputum production’, answer should be Yes.
  3. If there is any discordance between different sections of the note (e.g. ‘stable respiratory symptoms’ in the HPI but ‘new oxygen requirement’ in the assessment and plan), always use the findings in the assessment and plan section to guide your answer

Question 10: Has the patient had a respiratory related hospitalization based on the note? Answer only ‘Yes’ or ‘No’. Keep the following rules in mind:

- 1. Answer Yes if any hospitalization or admission is mentioned in the note and is due to any of the following criteria:
     1. Respiratory symptoms (e.g. cough, shortness of breath/dyspnea, increased or new oxygen requirement)
     2. Exacerbation of underlying lung disease
     3. Respiratory infection or illness
     4. Pleural effusion
     5. Pneumothorax
     6. Volume overload
     7. Left or right sided heart failure (CHF, CHF exacerbation, HFrEF or HFpEF exacerbation)
  2. Answer No if:
     1. There are no hospitalizations mentioned in the note (Note: emergency room or urgent care visits do not count as a hospitalization)
     2. Any hospitalization mentioned does not meet any of the criteria listed above

*Multi-class prompt:*

General instructions: You are a medical assistant that reads Notes from patient charts and answers questions. Please read the following patient Note and answer the questions based on the information explicitly stated within the Note and the question context.

Question 1: What type of ILD does the patient have as explicitly stated in the note? Please keep the following rules in mind:

1. First ensure that the patient has ILD. If the patient does not have ILD, then the answer should be none. The patient has ILD if any of the following are explicitly mentioned in the note:
   1. Diagnosis of ILD, even if the type is unclassified or pending further work-up (e.g. “ILD of uncertain etiology”
   2. Diagnosis of IPF, sarcoidosis/sarcoid, pulmonary fibrosis or fibrotic lung disease, NSIP, HP
   3. Patient is on nintedanib (ofev) or pirfenidone (Esbriet)
   4. Presence of specific CT scan findings such as reticulation, honeycombing, traction bronchiectasis, fibrosis, UIP, NSIP, or HP
   5. Pathology showing ILD, UIP, NSIP, or HP
   6. Of note, the patient does not have ILD if the diagnosis is explicitly ruled out (e.g. ‘no evidence of ILD’) or the patient has bronchiectasis alone without evidence of associated ILD
2. Answer IPF if:
   1. IPF is explicitly mentioned as the primary or most likely diagnosis being considered, even if it described as ‘likely secondary to’ or ‘probably caused by’ and
   2. No other ILD category is being considered
3. Answer CTD-ILD if:
   1. The patient has a definitive connective tissue disease and concurrent ILD (bronchiectasis alone does not count) and
   2. No other ILD category is being considered
4. Answer HP if:
   1. HP is explicitly mentioned as the primary or most likely diagnosis being considered and
   2. No other ILD category is being considered
5. Answer unclassifiable if:
   1. The note stated the ILD is unclassified or unclassifiable or
   2. Multiple ILD diagnoses are being considered, without one being more likely (e.g. IPF vs fibrotic HP)
   3. If there are multiple ILD diagnoses being considered answer unclassifiable followed by the diagnoses being considered. For example, if the note states “patient has IPF vs HP”, answer “unclassifiable, IPF, HP”
6. Answer other followed by the type of ILD if:
   1. The ILD listed in the note is not any of the above categories listed (IPF, CTD-ILD, HP, unclassified). For example, if the note states “patient has OP”, then the answer should be “other, OP”
7. If there is any discordance between different sections of the note, the assessment and plan section should be used to guide the final answer

**S2: Estimated Cost per Prompt-Note Combination**

**Assumptions:** ~3,500 input tokens (2,500-word note + question/prompt), ~10 output tokens (binary yes/no response)

**Example cost calculation:** OpenAI's GPT-4o current pricing is $2.50 per million input tokens and $10.00 per million output tokens. Therefore, the estimated cost would be approximately $0.0083 per inference per note.

- **Input cost:** 3,500 tokens ÷ 1,000,000 × $2.50 = **$0.00875**
- **Output cost:** 10 tokens ÷ 1,000,000 × $10.00 = **$0.0001**
- **Total:** $0.00875 + $0.0001 = **$0.008885**

| **Model** | **Input ($/1M tokens)** | **Output ($/1M tokens)** | **Cost per Prompt-Note Combination** | **Cost per Note (×10 runs x 10 prompts)** |
| --- | --- | --- | --- | --- |
| GPT-4o-mini | $0.15 | $0.60 | ~$0.0005 | ~$0.05 |
| GPT-3.5 Turbo | $0.50 | $1.50 | ~$0.002 | ~$0.18 |
| o3-mini* | $1.10 | $4.40 | ~$0.004 | ~$0.39 |
| Gemini 2.0 Pro | $1.25 | $5.00 | ~$0.004 | ~$0.44 |
| GPT-4o | $2.50 | $10.00 | ~$0.009 | ~$0.89 |
| Claude 3.5 Sonnet | $3.00 | $15.00 | ~$0.011 | ~$1.05 |
| Claude 3.7 Sonnet | $3.00 | $15.00 | ~$0.011 | ~$1.05 |
| o1-mini* | $3.00 | $12.00 | ~$0.011 | ~$1.05 |
| o1* | $15.00 | $60.00 | ~$0.053 | ~$5.25 |
| GPT-4 | $30.00 | $60.00 | ~$0.105 | ~$10.50 |

***Represent reasoning models with speculative costs. See description below.**

**Of note, Gpt-oss-20b and gpt-oss-120b are OpenAI open-weight models, and thus do not have standard API pricing.** **The reasoning models (o1, o1-mini, and o3-mini) have hidden “reasoning tokens” that also bill as output tokens, thus actual cost could be 2 to 10 times higher than shown. Therefore, costs for reasoning models are highly speculative and could significantly alter the economic feasibility of using these specific models at scale.**

These estimates are based on average token-to-word conversions and actual costs may vary slightly depending on the specific content and formatting of the document. Pricing also varies across models and is subject to change.

<https://platform.openai.com/docs/pricing>

(Accessed on 4/4/2026)
