# Supplementary figures and images for: Evaluation of Large Language Models for Structured Data Extraction From Interstitial Lung Disease Clinical Notes: Comparative Study
Source: J Med Internet Res. 2026 Jun 26;28:e90547. doi: 10.2196/90547 (PMC13354945; doi:10.2196/90547)

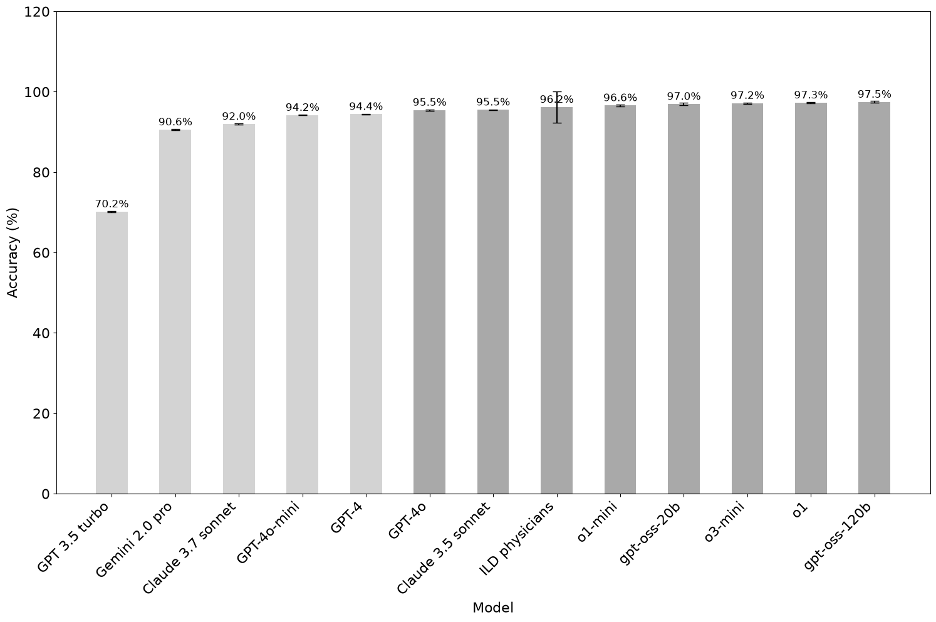

Supplement: Multimedia Appendix 3 [file jmir_v28i1e90547_app3.png]
